# Bone stimulators: Beyond the black box

**DOI:** 10.4103/0019-5413.50842

**Published:** 2009

**Authors:** Mohit Bhandari, Anil K Jain

**Affiliations:** *2309 Hoover Court, Hamilton, L7P 4V2, Ontario, Canada, India*; 1 *University College of Medical Sciences, and Guru Teg Bahadur Hospital, University of Delhi, New Delhi, India*

Each year in North America, approximately 6 million individuals suffer a fracture of whom 5–10% demonstrate delayed healing or nonunions.^1^,^2^ Extrapolating these statistics to India, we might expect 240 million fractures and 12 million nonunions annually. The developing countries also have nonunions and delayed unions following a failed previous implant surgery. Hence, there is a tremendous patient load needing bone stimulation. Besides that, India has a large number of septic nonunions.

Bone healing can be promoted by a number of novel therapeutics including bone stimulators. The most common bone stimulators include low-intensity pulsed ultrasound (LIPUS) and pulsed electromagnetic field therapy.^3^ The Food and Drug Administration, in 1994, approved the use of LIPUS for use in accelerating fresh fracture healing, and in 2000, for treatment of established nonunions.^4^ Similarly, electrical stimulation has been approved for the treatment of nonunions in the United States. Basic science research supports the use of LIPUS in signal transduction, gene expression, blood flow, and tissue modeling and remodeling.^5^ Multiple, small randomized trials have also supported the use of LIPUS.

The use of electric stimulation in the treatment of fractures nonunion dates back to the mid-1800s.^6^ In 1957, Fukada and Yasuda^7^ demonstrated a relationship between electricity and callus formation. Recent studies suggest that EMS impacts many cellular pathways, including growth factor synthesis, proteoglycan and collagen regulation, and cytokine production. These pathways may enable bone to respond to changing environments, ultimately stimulating the calcium/calmodulin pathway and thus enhancing bone healing.

Given the increased morbidity and costs associated with nonunions in our health care system, the identification of alternative bone healing therapies is warranted. LIPUS and electrical stimulation offer two noninvasive approaches to the promotion of healing of nonunions (and fresh fractures). The use of bone stimulators represents a multimillion dollar market globally; however, indications and evidence for each type of bone stimulator is not always transparent.

The current symposium provides an in-depth review of the current evidence for the two most common bone stimulators, LIPUS and electrical stimulation. We further provide new data for a lesser known bone stimulator, extracorporeal shock waves. Our symposium brings together experts in the field to provide insight into the basic cellular mechanisms of action as well as the clinical evidence base for fresh fracture healing and nonunions. The manuscript by George Methews extentively covers the newer developments in biology and biomechanics, fixation devices to affect the care of musculoskeletal injuries. Galkowski highlights the need for effective non-invasive therapies of bone stimulation to shorten the fracture healing time by decreasing the use of invasive procedures. Ultrasound therapy is covered in detail in three articles. The article by Gregory J. Della Rocca reviews the scientific evidence of cellular effects of ultrasound to accelerate normal fracture healing and promote fracture healing in a compromised tissue bed. The systematic reviews on LIPUS in fresh fractures and nonunions attempts to evolve the evidence of its use in the management of fresh fractures and nonunions. The article by Kuzyk RT and Schemitsch EH provides insight into the basic science of electrical stimulation. The article by Kooistra *et al*. is an overview on the currently available fundamental, preclinical, and clinical evidence on the biologic rationale and therapeutic efficacy of electrical stimulation devices in long-bone nonunions. A clinical series by Gupta *et al*. shows a clinical evidence of the use of pulse electromagnetic stimulation in nonunion tibial diaphyseal fractures. Petrisor *et al*. present a systematic review on extracorporeal shock wave therapy and its use in fracture management. The symposium ends with a systematic review on the economic evaluation of bone stimulation modalities and a discussion on available therapies, indications, and protocols for fracture healing in India.

Over the last several years, the concepts and ideas attributed to and labeled collectively as evidence based orthopedics have become a part of daily clinical lives, and clinicians increasingly hear about evidence based guidelines, care paths, and questions and solutions. This symposium of papers supports the new paradigm of evidence based orthopedics by providing an up-to-date critical appraisal of bone stimulators in 2009. Structured randomized controlled trials to compare the efficacy and safety, and a cost-effective analysis of bone stimulation therapies in comparison to invasive implant surgery are needed.
